# Economic burden of long COVID: macroeconomic, cost-of-illness and microeconomic impacts

**DOI:** 10.1038/s41533-025-00460-8

**Published:** 2025-11-21

**Authors:** Amit Bansal

**Affiliations:** 1 Influenza Centre, Department of Clinical Science, University of Bergen, Bergen, Norway; 2 Norwegian School of Sport Sciences, Oslo, Norway

**Keywords:** Diseases, Health care, Mathematics and computing, Medical research

## Abstract

Long COVID, defined by symptoms persisting three months post-SARS-CoV-2 infection, presents a significant global health and economic challenge, with global prevalence estimated at 36% (ranging from 1–92%). This brief communication consolidates current knowledge on its economic impacts, including macroeconomic, cost-of-illness, and microeconomic impacts, which are estimated at an average annual burden of $1 trillion globally and $9000 per patient in the USA, with some individuals covering substantial out-of-pocket expenses. Annual lost earnings in the USA alone are estimated at approximately $170 billion. Long COVID was associated with increased unemployment, financial distress, and work impairment for up to three years post-infection. This paper highlights discrepancies in impact estimation methodologies and calls for standardised metrics especially in emerging economies. Key research gaps include the absence of comprehensive longitudinal studies on individual and aggregated economic burden, specific long COVID phenotypes and biomarkers, and cost-effectiveness evaluations of interventions.

The COVID-19 pandemic, caused by the SARS-CoV-2 virus, has profoundly reshaped global health and economic landscapes. Beyond the acute phase of SARS-CoV-2 infection, a significant and increasingly recognised sequela is long COVID. The definition of long COVID varies by timeframes and organisation^1^. Based on a Delphi consensus in October 2021, the World Health Organization (WHO)^2^ defined long COVID as “the continuation or development of new symptoms 3 months after the initial SARS-CoV-2 infection, with these symptoms lasting for at least 2 months with no other explanation.”

Long COVID encompasses a wide array of debilitating symptoms affecting multiple organ systems, including severe fatigue, cognitive dysfunction (“brain fog”), cardiovascular, and neurological complications^3^. Given the multi-faceted nature of long COVID, which involves over 200 identified symptoms^4^ and various immunopathological pathways such as persistent viral presence^5–9^, viral reactivation^10–13^, and autoantibodies^13–17^, a single diagnostic code for long COVID may not fully encapsulate the condition’s complexity^18,19^. The chronic and often fluctuating nature of these symptoms has significant implications for individuals’ quality of life, their ability to work, and the functioning of healthcare systems worldwide. Consequently, understanding and quantifying the economic impacts of long COVID has become a critical imperative for policymakers, healthcare providers, and researchers globally, as nations grapple with a substantially affected population and the long-term societal fallout.

In the context of COVID-19 economic modelling, Chen et al. projected a cumulative gross domestic product (GDP) loss of approximately US$1.4 trillion by 2030 under an extreme scenario of unmitigated herd immunity^20^. When incorporating the value of a statistical life to account for mortality-related economic impacts, the estimated economic burden rises substantially — ranging from US$17 trillion to US$94 trillion over the next decade in the USA, with annualised fiscal impacts between 8 and 43%. Methodological choices further contribute to variability in the impact estimates. Bottom-up approaches, which aggregate individual patient-level impact, reported direct medical impacts ranging from US$ 1264 to US$ 79,315, while top-down models, based on national health expenditure or GDP metrics, estimated indirect impacts at approximately 11% of GDP in a systematic review and meta-analysis with studies from various countries including the USA (5), China (5), Spain (2), Brazil (2), South Korea (2), India (2), and one study each in Italy, South Africa, the Philippines, Greece, Iran, Kenya, Nigeria, and the Kingdom of Saudi Arabia^21^. These figures reflect differences in modelling frameworks rather than direct comparability across impact components.

The evidence base on economic impact of long COVID is organised across three analytical levels, highlighting methodological challenges and identifying critical gaps in the current evidence base. First, from a macroeconomic perspective, I examine prevalence, persistence, and GDP impacts, with particular attention to long-term morbidity and its implications for human and physical capital—especially labour market vulnerabilities in low- and middle-income countries (LMICs). Second, a cost-of-illness framework captures both direct and indirect impacts. Third, microeconomic dimensions are considered, including healthcare expenditure relative to income, declines in consumption, wage losses, familial impacts, and effects on education and human capital formation. Table 1 describes global financial burden of long COVID across differing healthcare settings and income group countries.

**Table 1:** Fiscal impact of long COVID.

Region	Macroeconomics*		Cost-of-illness	Microeconomics
	Prevalence with 95% confidence interval (95% CI)	Key impacts, including GDP losses		
Africa	53% (38–67%), based on a systematic review^22^	NA^$^	In South Africa, mean direct impacts of COVID-19 were US$ 1908 and daily economic impact related was US$ 184–1327 per patient (depending on severity)^21,70^	Substantial due to informal labour markets and limited social safety nets^21^
Asia	35% (25–46%)^22^	According to the Economist Group, long COVID attributable economic impacts may have exceeded US$72.2 billion in Japan and US$24.4 billion in Saudi Arabia^1^ in 2024.	NA^$^	In 2024, long COVID may have led to over 1.8 billion lost work hours in Japan and 670.7 million in Saudi Arabia^1^.
Europe	39% (31–48%)^22^	In the UK, the mean productivity loss per incident infection between 2022 and 2023 was estimated at £10,929, with a national extrapolation of £5.7 billion (95% CI: £3.8–£7.6 billion)^71^. In 2024, the Economist Group estimated associated economic impacts were exceeding US$21 billion in France and US$7.8 billion in Spain1.	In the UK, £705 median annual healthcare impact for long COVID cohort^38^. In France, €823 total annual per-patient impact for children with long COVID^37^	The Economist Group estimated over 295 million lost work hours in France and 167.8 million in Spain in 2024 due to long COVID^1^. This can potentially increase financial distress and sustained work impairments.
North America USA	30% (24–38%)^22^ (North America). In the USA, 29% (21–37%)^22^	In the USA, $6.4 billion productivity loss^63^; potentially $170–230 billion lost earnings annually^32–36^. In Canada, the total burden on the healthcare system from long COVID was estimated to be between CAD 7.8 and CAD 50.6 billion up to spring 2023, with unvaccinated individuals having higher impacts^72^.	Estimated $9000 per individual with long COVID annually in the USA (based on ME/CFS analogy)^30,31^. Long COVID patients had 1.5–1.8 times higher medical impacts than controls over 6 months in 2020 (CDC study)^41^.	In 2021, among individuals with long COVID, 10% stopped working temporarily due to symptoms, and a substantial proportion reported reduced working hours (a USA report by the Federal Reserve^73^), though precise estimates are still unclear.
South America	51% (35–66%)^22^	The Economist Group estimated that long COVID may have led to a potential economic impact exceeding US$11 billion in Brazil (assuming only 3% of the total population with long COVID). Nevertheless, rigorous economic analyses are required to comprehensively assess the extent of this impact^1^.	NA^$^	In 2024, long COVID may have led to a loss of over 803 million work hours in 2024 in Brazil^1^.
Oceania	18% (18–19%)^22^	In Australia, the associated GDP loss was projected at $9.6 billion (95% CI: $4.7–15.2 billion) in 2020–2021, equivalent to 0.5% of GDP^27^. In 2022, the Australian economy faced a potential burden of between $1.7 billion and $6.3 billion, equivalent to 0.07–0.26% of its GDP^74^.	NA^$^	Based on the same modelling study, labour loss due to long COVID in 2022 amounted to 102.4 million hours (95% CI: 50.4–162.2 million), representing 0.48% (95% CI: 0.24–0.76%) of total hours worked in 2020–2021 in Australia^27^. Labour market data indicate that, between February and June 2023, an estimated 25,000–103,000 additional working-age Australians reported long-term illness-related work incapacity, exceeding projections based on pre-COVID-19 trends^74^.
Global	Pooled estimate: 36% (33–40%), varies widely by study and sub-populations^22^. Likely 2–7% based on expert opinion^1^.	US$1 trillion/year attributable to long COVID, which represents around 1% of the global economy^28–31^.	NA^$^	Limited long COVID global data: however, substantial portion of long COVID sufferers stopped working over 6 months post-infection^61^.

^*^The economic burden in this context encompasses three dimensions: (1) a macroeconomic perspective, which considers factors such as prevalence, the persistence of symptoms, and potential declines in GDP; (2) cost-of-illness, including both direct medical and indirect impacts; and (3) microeconomic aspects, such as healthcare expenditures as a proportion of household income, reductions in consumption levels, wage losses, financial strain on families, and implications for education and human capital accumulation—for instance, diminished investment in education.

^$^NA: Not available. Limited long COVID, region-specific, data attributing to its potential economic burden.

## Macroeconomic perspective

Experts broadly concur on a more conservative prevalence figure of 2–7% in some form^1^. However, the global pooled prevalence of long COVID amounted to 36%, based on a systematic review of 144 studies published up to 2024, with rates remaining stable over time and a variation largely due to heterogeneous study populations and subjective diagnostic criteria^22^. The estimated pooled prevalence was 38% in 2021 (ranging from 10–62%), 37% in 2022 (ranging from 1–92%), and 37% in 2023 (ranging from 6–87%)^22^. A higher prevalence was observed in patients who required hospitalisation, as well as in females and adults^22^. This prevalence estimate decreased slightly to 34% in 2024 (ranging from 3–80%). A global modelling study^23^ estimated a 7-fold increase in the global median prevalence of long COVID between 2020 and 2022, largely due to cumulative infections.

Long COVID presents with comparable symptoms and functional impact across global subpopulations, though prevalence and severity may vary by population^24^. Estimated prevalence of long COVID is higher in high-income countries (HICs); however, its burden may be greater in LMICs due to limited support systems^24^. Geographically, the prevalence was found to be highest in Africa and South America, followed by Europe, Asia, North America, and Oceania^22^. In France, the prevalence of long COVID was estimated at 4.0% (95% confidence interval, 95% CI: 3.6–4.5) in the general population, and 8.0% (95% CI: 7.0–8.9) among individuals with a confirmed infection^25^. A USA study reported that 14% of adults had ever experienced long COVID, with about half still symptomatic, with data from nearly half a million Americans in the period June 2022–December 2022 in the US Census Bureau’s Household Pulse Survey^26^. An Australian modelling study projected that long COVID cases following a single infection in 2022 would peak in September, affecting 310,341–1,374,805 individuals (1.2–5.4% of Australians), declining to 172,530–872,799 (0.7–3.4%) by December 2024, including 7902–30,002 children aged 0–4 years (0.6–2.2%)^27^. Despite this, there is still an unmet clinical need to accurately diagnose the individuals with long COVID with a heavy reliance on clinical diagnosis and no approved objective diagnostic tests, leading to potential bias in prevalence estimates.

From a macroeconomic standpoint, the burden of long COVID is material and persistent over years^22,23^. Long COVID was estimated to affect 400 million individuals globally, though this figure is arguable, with a potential annual economic burden approaching $1 trillion—approximately 1% of global GDP based on extrapolated models^28^. Moreover, Economist David Cutler estimated model-based economic loss of approximately $3.7 trillion attributable to long COVID^29–31^. Most of this loss, 59%, is attributed to a decrease in quality of life, while the remaining the losses are due to reduced earnings and increased medical expenses^29^. Brookings Institution report^32^, published in 2022, analysed information from the Household Pulse Survey on the social and economic impacts of long COVID^33^. It estimated 2–4 million working-age Americans (out of 16 million with long COVID) were unemployed or had left the labour force due to the condition, resulting in an estimated $170 billion in lost earnings annually (and potentially as high as $230 billion), or nearly 1% of the US GDP^32–36^. This report was not peer-reviewed and should be interpreted with caution. In Australia, the projected average economy-wide loss in 2022 was approximately $9.6 billion, equivalent to around one-quarter of the nation’s real GDP growth for that year^27^. These figures vary widely due to differences in modelling approaches, definitions, and data sources. Notably, most studies rely on cross-sectional data from HICs, limiting generalisability. Data from LMICs are scarce and are prone to misclassification when diagnostic infrastructure is limited.

## Cost‑of‑illness framework (direct medical and indirect impacts)

Direct medical impacts encompass expenses related to medical care, including general practitioner visits, specialist consultations, diagnostic tests (e.g., imaging, blood tests), hospitalisations, rehabilitation services (e.g., physiotherapy, occupational therapy), and prescription medications. The chronic and multi-systemic nature of long COVID often necessitates extensive and multidisciplinary care, driving up healthcare expenditure. The direct medical impacts associated with the treatment of long COVID have not yet been comprehensively estimated; however, projections have been made for comparable conditions, especially in HICs. Economist David Cutler has suggested that, assuming treatment parallels that of myalgic encephalomyelitis/chronic fatigue syndrome, the average annual impacts per patient could be approximately $9000 in the USA, with some individuals potentially bearing nearly half of these expenses out-of-pocket (model-based estimates)^30,31^. However, impacts likely vary widely by country, insurance status, and symptom severity. A study in France found that children with mild-to-moderate long COVID incurred an incremental impact increase of €98 per patient per year compared to those without long COVID, with total annual per-patient impact reaching €823 in the first year alone, indicating a non-negligible burden on national health insurance^37^. In the UK, a large study involving 282,080 adults with long COVID found that the median annual healthcare impact for long COVID cohort was £705, substantially higher than observed in pre-long COVID (£294), COVID-19 only (£447), pre-pandemic (£306), and contemporary non-COVID-19 (£350) cohorts^38^. This significant increase in healthcare demand poses a global challenge for health service resourcing.

Beyond direct medical impacts, long COVID incurs broader societal, out-of-pocket medical and hidden impacts. These include fully self-funded treatments, contributions to public healthcare, insurance co-payments, and the burden on informal caregivers, who often provide extensive support to affected individuals, leading to their own reduced work hours or experience heightened stress. Intangible impacts —such as diminished quality of life, reduced social participation, and psychological distress among individuals with long COVID and their families—are substantial yet frequently undocumented (see microeconomic impacts). While difficult to quantify monetarily, these impacts represent a profound impact on societal well-being. The varied clinical presentation of long COVID further complicates the impact estimation, as different symptom clusters may necessitate distinct diagnostic and therapeutic pathways, each with its own associated impacts.

An individual’s clinical background and incremental impacts are likely attributable to heightened utilisation of healthcare resources—most notably inpatient hospital admissions—and to the ongoing management of a broad spectrum of newly emerging or worsening comorbid conditions. A cohort study of post-hospitalised COVID-19 patients found limited recovery in symptoms, function and fatigue at six months, with some reporting new health issues during follow-up^39^. A matched cohort study^40^ in the UK found that, in the 12 months post-diagnosis, individuals with long COVID had significantly higher healthcare use (odds ratio = 8.3; relative risk = 1.5), averaging 30 healthcare visits per year versus 16 visits in comparators. Long COVID group was more likely to incur overheads, with annual expenditures 44% higher (£2562 vs £1527)^40^. While specific medication impact for long COVID is still being studied, the overall burden is high due to prescriptions for managing symptoms and comorbidities. A matched cohort study of privately insured individuals in the USA found that direct medical impacts were 1.5–1.7 times higher for those with long COVID than for controls over 3- and 6-months post-diagnosis^41^.

Interventions against long COVID are still evolving; however, as of now, current therapeutic strategies for long COVID remain largely supportive and symptomatic, with no specific non-drug or drug interventions proven as definitively effective in large-scale clinical trials. Recent studies suggest that long COVID symptom improvement may occur in some patients after cognitive behavioural therapy and multidisciplinary rehabilitation^42–45^, noninvasive brain stimulation^46–48^, antiviral therapy^49^, hyperbaric oxygen therapy^50,51^, palmitoylethanolamide and luteolin (PEA-LUT) use^52–54^; however, no precise cost-effectiveness estimate exists. Moreover, higher vaccination rates significantly reduce prevalence (by ~21% in the USA adults and ~16% globally)^23^. The cost-effectiveness of vaccines in preventing long COVID is typically influenced by the circulating SARS-CoV-2 variant, the timing of vaccine administration, and the characteristics of the population at highest risk^55,56^. A supervised online rehabilitation programme yielded an incremental cost-effectiveness ratio of £11,941 per quality-adjusted life year (QALY) over 12 months, indicating cost-effective care^42^. Cognitive behavioural therapy, physical and mental health rehabilitation, and certain aerobic exercise programmes probably improve long COVID symptoms such as fatigue, function, and quality of life (moderate certainty)^43^, potentially reducing its economic impact. Outpatient metformin use reduces long COVID risk over 6–10 months post-infection, according to a large cohort study^57^ and phase 3 clinical trial^58^, suggesting high economic value given its low impact, though formal evaluations are pending. More robust economic evaluations are needed, especially in LMICs.

## Microeconomic perspective

At the microeconomic level, long COVID was associated with higher unemployment, reduced hours^59^, and financial distress, persisting up to 3 years post‑infection and partially mitigated by prior vaccination^60^. Microeconomic impacts including lost productivity and workforce participation are important to estimate complete economic burden of long COVID. The most substantial economic burden of long COVID often stems from its impact on workforce participation and productivity. Many individuals with long COVID experience debilitating symptoms that prevent them from returning to their previous employment, reduce their working hours, or diminish their capacity to perform work-related tasks effectively, leading to lost wages for individuals and reduced output for the economy. Globally, a study spanning 56 countries revealed that nearly half (45%) of 1700 respondents were ill for over 28 days with suspected and confirmed COVID-19 reported reduced work schedules, with 22% (839 individuals) not working more than six months after falling ill^61^.

A survey of over 15,000 adults with test-confirmed COVID-19 at least 2 months prior found that individuals with self-reported long COVID symptoms were more likely to be unemployed (12.3% compared to 8.7% of those without)^35,62^. Similarly, a recent PLOS One study on absenteeism found that US employees with long COVID missed, on average, eight days within a calendar year, compared to four days for those without long COVID, translating to an estimated 23 million lost workdays annually and a productivity loss of approximately $6.4 billion^63^. This disproportionately affects women and workers in low-wage, face-to-face industries^59,64^. The cumulative effect of these individual impacts translates into a substantial loss of GDP and labour supply for nations worldwide. However, the persistence of such economic impact over time remains uncertain, as individuals with long COVID tend to recover over time and longitudinal data on long-term productivity loss are still limited.

A multicentre cohort study^60^ found that worse financial outcomes, such as financial distress and impaired ability to work, persisted for up to 3 years after SARS-CoV-2 infection among US adults. While rates of return to work varied significantly, from 10–100%, prior vaccination was found to mitigate the work impairment. Specifically, vaccination was associated with 29% lower odds of work impairment and a 34% lower incidence of impairment in non-work activities; however, vaccination was not linked to a lower severity of financial distress^60^. Moreover, cross-sectional analyses of data from the 2022 National Health Interview Survey^65^ found that adults with long COVID experienced significantly greater financial stress than individuals with other chronic conditions. The odds were higher compared to those with epilepsy, dementia, cancer, and respiratory or cardiovascular diseases. Financial hardship in this group was associated with female sex, age under 65 years, lack of health insurance, residence region, food insecurity, fatigue, depression symptoms, emergency department visits, arthritis, cardiopulmonary conditions, and limitations in social activity^65^.

A disaggregated analysis at income and education levels linked long COVID to an increase in financial hardship of 1–11 percentage points across nearly all income and education brackets, disproportionately affecting lower-income groups (income-to-poverty ratio below 2), with 6–20% of this association mediated by employment loss or reduced work hours^59^. Furthermore, a qualitative study identified school attendance limitation and functional impairment in children and young individuals with long COVID^66^.

Emerging evidence suggests that the economic implications of long COVID are profound and multifaceted, necessitating a comprehensive understanding to inform global policy and resource allocation. The long-term implications for mental health services, social support systems, and disability benefits may also emerge as significant areas of concern, representing substantial future liabilities. One notable area of controversy revolves around the methodological approaches to estimating the economic impact of long COVID. Divergences arise in how cases are defined, the inclusion criteria for various impact components, and the time horizons over which impacts are projected. Some studies focus solely on direct healthcare impacts, while others incorporate a broader range of indirect impacts, leading to varying aggregate estimates^21^. There is also ongoing debate regarding the true global prevalence of long COVID, which directly biases the scale of estimated impacts. Furthermore, the attribution of impacts specifically to long COVID, as opposed to pre-existing conditions or other confounding factors, presents a methodological challenge. The heterogeneity of long COVID symptoms also makes standardised impact estimation difficult, as different symptom clusters may incur vastly different expenses.

Despite growing attention, several critical research gaps remain in understanding the global economic impacts of long COVID. Firstly, there is a dire need for comprehensive longitudinal studies that track the economic impact on individuals and healthcare systems over extended periods, ideally across diverse geographical and socioeconomic contexts. Most existing data are cross-sectional or short-to-medium term, failing to capture the chronic and fluctuating nature of the condition. Secondly, more granular analyses are required to understand the economic burden associated with specific long COVID phenotypes or symptom clusters. For example, the impacts associated with neurological long COVID (e.g., severe cognitive impairment) may differ significantly from those primarily involving fatigue or respiratory symptoms. Thirdly, there is a significant gap in research evaluating the cost-effectiveness of various interventions for long COVID, including multidisciplinary rehabilitation programmes, pharmacological treatments, and mental health support. Understanding which interventions offer the best value for money is crucial for efficient resource allocation within constrained healthcare budgets globally. Finally, robust data collection and harmonisation across different healthcare settings and countries are essential to allow for meaningful comparisons and the development of best practices. The lack of standardised diagnostic codes, evidence-based biomarkers, and reporting mechanisms for long COVID also hinders accurate and comparable data aggregation for estimating prevalence and economic losses.

Future efforts to mitigate the escalating global economic consequences of this chronic condition must focus on robust surveillance, integrated care pathways, sustained research investment, and equitable policies. Specifically, future developments in addressing the economic impacts of long COVID may focus on the following actionable areas:

1. Enhanced global surveillance and data harmonisation: Establishing robust national and international surveillance systems for long COVID, particularly in diverse socioeconomic and geographic contexts, will be paramount to accurately track prevalence, incidence, and the associated economic burden over time with an agreed definition of long COVID. This necessitates standardised diagnostic criteria and data collection protocols, fostering international collaboration to create comparable datasets.
2. Development of integrated care pathways: The implementation of integrated, multidisciplinary care pathways for long COVID patients is crucial to optimise resource utilisation and mitigate escalating healthcare impacts. These pathways should aim to reduce diagnostic delays, streamline referrals, and ensure continuity of care, potentially drawing lessons from existing models for other chronic conditions.
3. Targeted research investment in interventions and LMICs: Sustained and targeted investment in research into the epidemiology, pathophysiology, cost-effective treatments, and preventative strategies for long COVID is vital. Breakthroughs in these areas have the potential to significantly reduce the long-term economic burden by enabling faster recovery and reduced symptom severity. This includes funding for biomedical research and clinical trials focusing on antivirals, immune modulation, rehabilitation, and tissue damage^67^. Although direct evidence from LMICs is limited, existing economic fragilities—such as high debt and constrained fiscal space—heighten vulnerability to the long-term labour impacts of chronic illness like long COVID. Strengthening primary care, expanding rehabilitation access, and targeted investing in health expenditure tracking are essential to ensure equitable recovery and resilience.
4. While advanced modelling techniques exist, the current priority is to raise awareness of the economic implications of long COVID and encourage foundational modelling efforts with better data collation. Basic approaches—such as cost-of-illness projections (disease burden and differing long COVID subpopulations), cost-effectiveness analyses of interventions (due to changes in treatment paradigms), and labour market impact assessments (human capital/production function modelling. e.g., flexible and remote work arrangements)—are sufficient to inform policy and guide resource allocation. These methods, often applied successfully to other chronic conditions, remain underutilised in the context of long COVID. Countries should optimise the use of their limited data resources, particularly by harnessing the potential of existing disease registries. Future work should focus on adapting existing frameworks and involve micro-simulation models to project future burdens, expenses, and potential savings from interventions, drawing on methodologies used for other non-communicable diseases^1,68^.
5. Policy initiatives for workforce support: Governments and employers must develop proactive policies to support individuals with long COVID in the workforce. This includes flexible work arrangements, disability benefits, rehabilitation programmes, and access to mental health support to reduce absenteeism and presenteeism and facilitate return to work, where possible. Policies that enable remote work including individuals with long COVID or related disability in a leadership position, where feasible, can also increase labour force participation among those with disabilities, including long COVID^34,69^.
6. Learning from other chronic conditions: It is reasonable to use the term chronic post-viral syndromes to capture the disease complexity and to help uncover the underlying cause of other life-limiting post-viral infections, such as Myalgic Encephalomyelitis/Chronic Fatigue Syndrome (ME/CFS) and Multiple Sclerosis. The economic modelling and management strategies for other chronic post-viral syndromes or debilitating conditions (e.g., ME/CFS) can provide valuable insights for projecting and mitigating the long-term economic burden of long COVID^68^.

In conclusion, long COVID represents a significant global health economic challenge. The impacts are borne across healthcare systems, individual households, and national productivity, with hidden out-of-pocket expenses further exacerbating the burden. While initial estimates underscore the scale of the problem, significant evidence gaps remain, particularly regarding longitudinal data, LMIC contexts, the specific expenses within diverse long COVID phenotypes, and the cost-effectiveness of interventions. Addressing these gaps, alongside the development of robust global surveillance, integrated care pathways, and sustained research investment coupled with equitable workforce policies, will be critical in mitigating the long-term economic ramifications of this pervasive and debilitating post-viral condition. The long-term prosperity and health of the global population are inextricably linked to effectively managing the economic burden of long COVID.

## Methods

This Brief Communication was conducted through a focused rapid narrative literature search aiming to synthesise current evidence on the economic impacts of long COVID. The primary search strategy involved querying academic databases, predominantly MEDLINE Ovid and Embase Ovid, for peer-reviewed articles published in the English language up until 19 September 2025. Keywords used in various combinations included “long COVID,” “Post-Acute Sequelae of SARS-CoV-2 infection,” “PASC,” “economic cost,” “economic burden,” “healthcare costs,” “productivity loss,” and “societal cost.” Additional relevant articles were identified through snowballing from the reference lists of retrieved papers and authoritative reports from international organisations and research institutions.

This Brief Communication included studies that provided quantitative data on direct and indirect economic impacts of long COVID across different countries, with a particular emphasis on recent findings. Exclusion criteria comprised non-peer-reviewed literature, preprints, and unpublished data. Studies focusing solely on acute COVID-19 without reference to long-term sequelae were also excluded from the primary analyses.

The identified literature was critically appraised for relevance, with a focus on studies that quantified economic parameters, discussed methodological challenges, or proposed future research directions. The synthesis of findings informed the thematic organisation of the review, addressing direct, indirect, and broader societal impacts, as well as outlining current controversies, research gaps, and potential future developments in the field.

The rapid review approach, while enabling timely synthesis, is inherently limited by its scope and potential for selection bias. The Economist and Brookings Institution reports are not peer-reviewed^1,32^, and should be interpreted with caution. Moreover, the heterogeneity in study designs, impact components, lack of evidence-based biomarkers with self-reported diagnosis, differing definitions of long COVID, and national contexts complicates direct comparisons and generalisability. Most data are from HICs and cross-sectional studies, limiting causal inference, and are prone to geographic and socioeconomic bias. Additionally, many studies cited do not adjust for pre-existing conditions, which could inflate impact estimates attributed to long COVID. These limitations underscore the need for more standardised and longitudinal economic evaluations of long COVID.

## Data Availability

No new original data is generated.
